# Case Report: Tube perforation after bleb reformation after PreserFlo MicroShunt implantation

**DOI:** 10.3389/fopht.2026.1749614

**Published:** 2026-06-03

**Authors:** Masashi Nishigaki, Atsuya Miki, Chiaki Nogawa, Motohiro Kamei

**Affiliations:** 1 Department of Ophthalmology, Aichi Medical University, Aichi, Japan; 2 Aichi Medical University Eye Center, Aichi, Japan; 3 Department of Myopia Control Research, Aichi Medical University, Aichi, Japan

**Keywords:** glaucoma, PreserFlo MicroShunt, prostaglandin-associated periorbitopathy, tube exposure, tube perforation

## Abstract

**Purpose:**

To describe a rare case of tube perforation after PreserFlo MicroShunt (PMS) implantation and subsequent bleb revision, emphasizing the potential mechanisms involved and the strategies employed for clinical management.

**Observations:**

An 84-year-old woman with bilateral exfoliation glaucoma underwent PMS implantation in her left eye due to uncontrolled intraocular pressure (IOP) despite receiving maximum medical therapy. The patient had a history of multiple ocular surgeries and long-term use of glaucoma eye drops. The initial surgery was uneventful; however, the patient developed hypotony and choroidal detachment after stent removal, prompting a second procedure for stent reinsertion. Subsequently, a postoperative conjunctival leak developed, and petrolatum-based ophthalmic ointment was used; however, the leakage did not improve, leading to a third surgery. During this procedure, a conjunctival defect and a 0.5-mm tear posterior to the fin of the PMS tube were identified. The device was removed, and a new PMS tube was implanted in a different quadrant. The IOP stabilized postoperatively, with no recurrent leakage or hypotony.

**Conclusions and importance:**

This is a rare case report of PMS tube perforation with a conjunctival hole. Potential contributing factors included chronic conjunctival inflammation from long-term use of glaucoma eye drops, previous ocular surgeries, and prostaglandin-associated periorbitopathy. These conditions may have resulted in tissue thinning and mechanical stress, leading to tube exposure and perforation. In addition, loss of the conjunctival barrier and subsequent exposure to petrolatum-based ophthalmic ointment may have compromised the structural integrity of the PMS, potentially leading to perforation due to continued eyelid friction. A careful preoperative assessment of the ocular surface and periocular anatomy is essential for identifying high-risk patients. Furthermore, if the PMS becomes exposed postoperatively, the use of petrolatum-based ophthalmic ointment should be avoided.

## 1 Introduction

The PreserFlo MicroShunt (PMS) (Santen Inc., Osaka, Japan) is a minimally invasive glaucoma drainage device designed to facilitate aqueous humor flow from the anterior chamber to the subconjunctival or sub-Tenon’s space. The microshunt, which is made of poly(styrene-block-isobutylene-block-styrene) (SIBS), an inert, biocompatible material, is 8.5 mm long and has a 70-μm lumen. Unlike traditional filtering surgeries, PMS implantation does not require a scleral flap, sclerostomy, or peripheral iridectomy, reducing the risk of postoperative inflammation and complications.

Previous studies have demonstrated that PMS implantation is associated with fewer postoperative interventions and a lower incidence of complications compared to trabeculectomy (1).

However, several complications have been reported after PMS implantation, including tube exposure. Exposure of the tube shunt device through the conjunctiva is a serious concern that increases the risk of infection (2, 3). Reported risk factors for tube exposure include the absence of Tenon’s capsule, blepharitis, and a history of multiple ocular surgeries (2, 4, 5).

Here, we report a case of tube exposure and associated tube perforation after bleb reformation after initial PMS implantation. We believe that this report will be helpful for the identification of possible tube damage and to determine the measures to implement in these cases.

## 2 Case description

An 84-year-old woman with bilateral exfoliation glaucoma presented with medically uncontrolled intraocular pressure (IOP) in her left eye despite maximal medical therapy (bimatoprost, timolol 0.5%/brinzolamide, and ripasudil/brimonidine 0.1%). The patient had deepening of the upper eyelid sulcus (DUES), conjunctival injection, and eyelid hardening. The left eye had undergone multiple surgeries: cataract surgery via a superior corneal incision 10 years before at an external hospital; vitrectomy for vitreous hemorrhage 6 years before with port incisions at the 2, 4, 8, and 10 o’clock positions; and ab interno trabeculotomy using a Kahook Dual Blade via a temporal corneal incision 3 years before. At presentation, the best-corrected visual acuity (BCVA) in the left eye was 0.9, and the IOP was 34 mmHg. The axial length was 22.5 mm. Visual field testing showed a mean deviation of -14.13 dB in the left eye. A PMS implantation was scheduled for the left eye.

Under local anesthesia, an initial PMS implantation was performed according to the standard procedure. Briefly, 0.04% mitomycin-C was injected subconjunctivally in the superior nasal quadrant using a 30-gauge needle inserted through the conjunctiva at a site approximately 12 mm posterior to the limbus, and it was allowed to soak for 3 min, followed by thorough irrigation. This area partially overlapped with the site of the previous scleral incision for cataract surgery and was close to the site of the 10 o’clock port incision from the previous vitrectomy. Previous studies comparing the efficacy and safety of soaking MMC in a sponge versus conjunctival injection have reported no significant differences. (6) After fornix-based dissection, a scleral tunnel was created 3 mm posterior to the limbus using a double-step knife. A PMS was inserted into the anterior chamber, and aqueous outflow from the posterior end was confirmed. A 10–0 nylon suture was inserted as a stent in the tube to prevent early postoperative hypotony. Tenon’s capsule and the conjunctiva were closed together as a single layer, and dexamethasone was injected subconjunctivally. The surgery was completed uneventfully. Postoperatively, the patient received topical gatifloxacin four times and dexamethasone six times daily.

**Figure 1:**
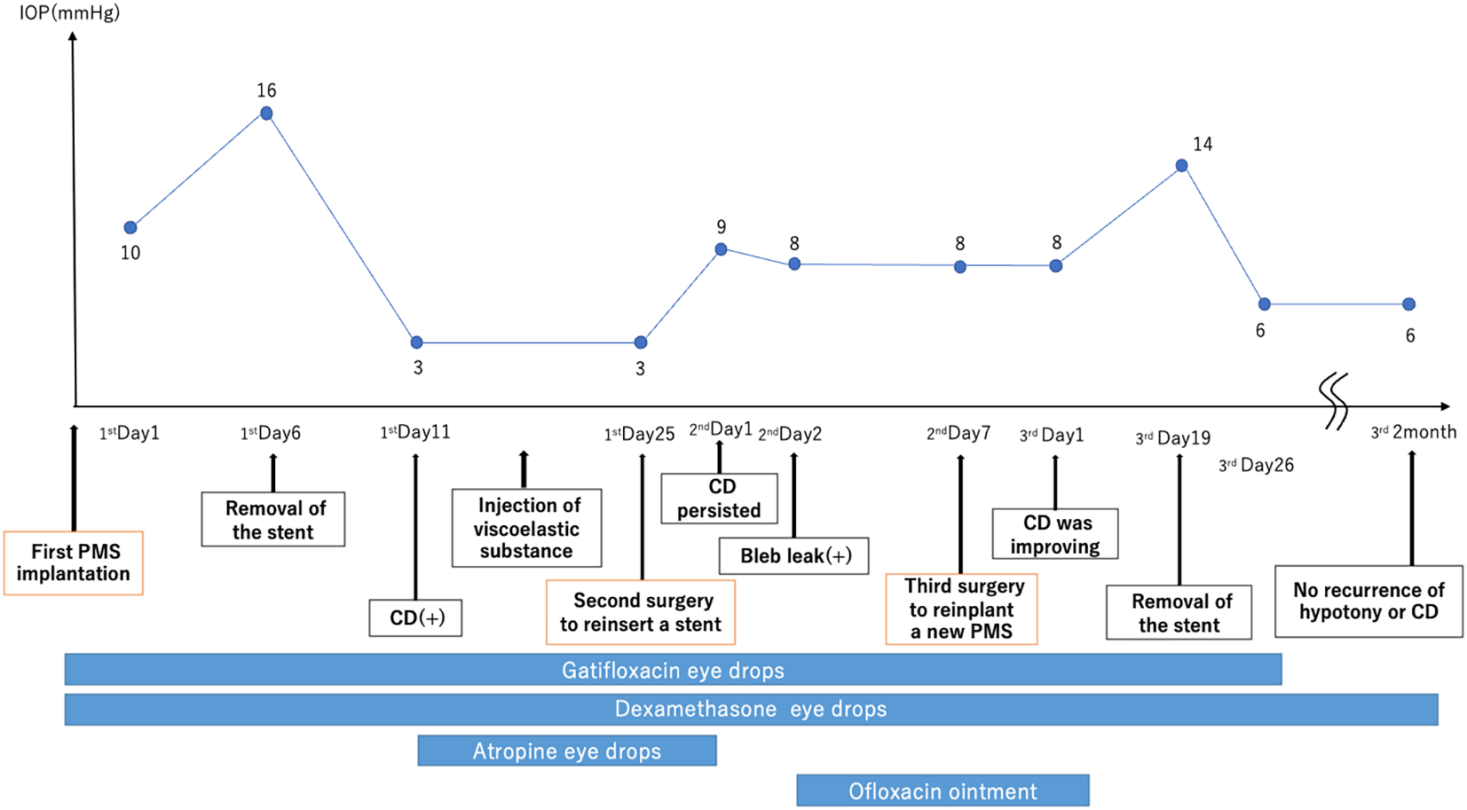
Postoperative course. CD, choroidal detachment. PMS, PreserFlo MicroShunt.

The postoperative course is shown in **Figure 1**. On postoperative day 1, a functional bleb and a deep anterior chamber with mild hyphema were observed, and the IOP decreased to 10 mmHg. The hyphema resolved gradually. By day 6, the IOP increased to 16 mmHg, and the stent was removed.

On postoperative day 11, the IOP decreased to 3 mmHg. A Seidel test was performed at that time and was negative. Although the anterior chamber remained deep, a prominent choroidal detachment (CD) was confirmed by fundus examination. CD was present in all quadrants and was close to a kissing choroidal detachment. Although atropine was added, the condition worsened; therefore, on postoperative day 18, Healon V^®^ was injected into the anterior chamber. On postoperative day 20, the IOP was 6 mmHg, and the CD showed some improvement; however, by postoperative day 25, the IOP had decreased again to 3 mmHg, and the CD had worsened. Therefore, a second surgery for stent reinsertion was scheduled. Throughout the procedure, the filtration bleb appeared flat or markedly thinned.

The previous surgical site was reopened. The PMS tube was exposed, and a 10–0 nylon suture was inserted from the tube lumen into the anterior chamber. A partial defect in the Tenon’s capsule was observed, which was located near the corneal limbus corresponding to the site of filtration bleb formation and was distant from the PMS outlet. The remaining Tenon’s tissue was re-approximated as much as possible and closed together with the conjunctiva as a single layer. We confirmed that there was no leakage from either the tube or the conjunctiva at the end of the surgery. The conjunctival tissue and Tenon’s capsule appeared thinner than during the initial procedure.

On postoperative day 1 from the second surgery, the IOP was 9 mmHg, with a deep anterior chamber and functioning bleb. The CD persisted but was improving. However, on postoperative day 2 from the second surgery, a bleb leak from the conjunctiva above the tube was observed. The filtering bleb was flat and ischemic. Because the leakage did not change after prescribing ofloxacin ointment four times daily, a third surgery was performed.

**Figure 2:**
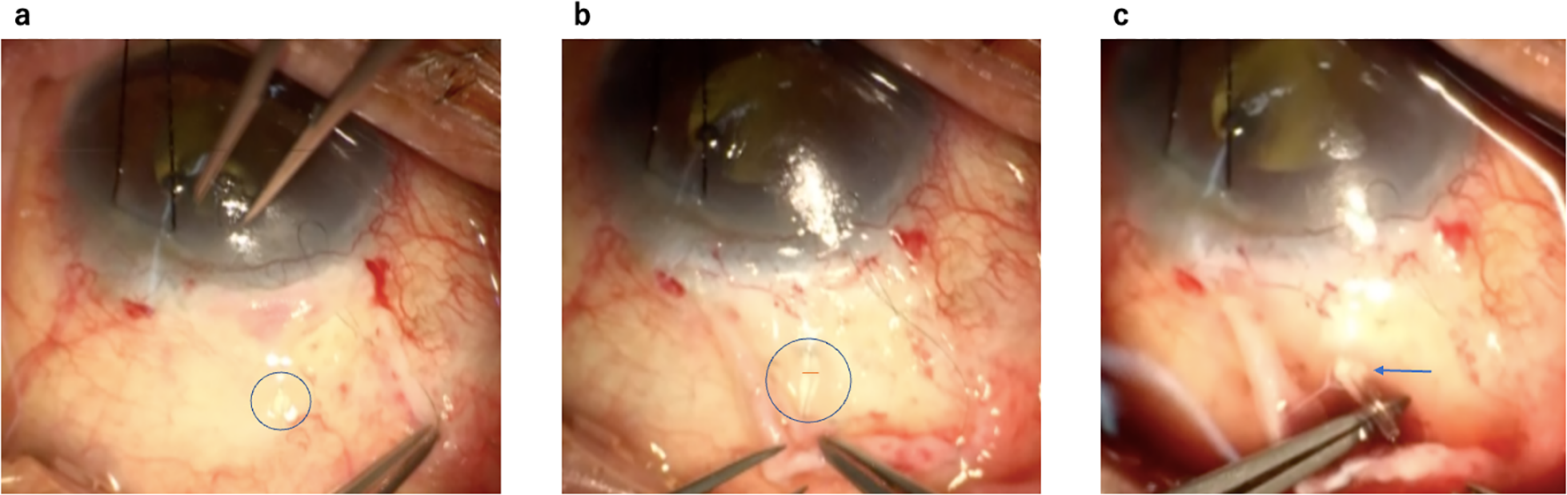
Images from the third surgery. **(a)** A conjunctival hole with the tube exposed is visible. **(b)** After exposing the tube through a conjunctival incision, a perforation was found in the tube. This is indicated by a red line. **(c)** Leakage was observed at the perforation site(arrow). Leakage consisted of aqueous humor that had filtered from the anterior chamber into the PMS.

**Figure 3:**
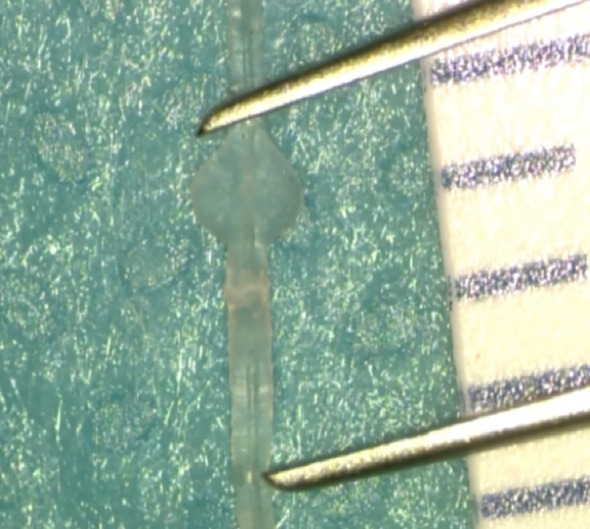
The removed tube. A tear was observed 0.5 mm posterior to the fin.

A hole was present in the conjunctiva above the tube, through which aqueous humor was leaking. The conjunctiva was reopened to expose the PMS tube, and a tear 0.5 mm posterior to the tube fin was seen, indicating leakage at that site (**Figures 2**, **3**). The location of the tear was immediately beneath the conjunctival defect. The microshunt was removed. The thin Tenon’s capsule was advanced anteriorly to cover the scleral incision site. The conjunctiva was closed at the limbus. As no leakage was observed after suturing, the conjunctival defect was left unsutured. A new microshunt and an intratube nylon stent were inserted from the superotemporal quadrant using the same technique as the initial surgery.

On postoperative day 1 from the third surgery, the IOP was 8 mmHg, with a deep anterior chamber and well-formed bleb. The CD was improving. On postoperative day 19 from the third surgery, the IOP increased to 14 mmHg, the CD disappeared, and the stent was completely removed. Subsequently, the IOP decreased to 6 mmHg on postoperative day 26 from the third surgery. The Seidel test remained negative from the third surgery onward.

Two months after the third surgery, the BCVA was 0.3, and the IOP remained at 6 mmHg, without recurrent hypotony or CD.

## 3 Discussion

This is a case of partial microshunt exposure and subsequent perforation of the exposed microshunt. Several reports of microshunt exposure have been published (7–10). Tanito et al. reported a case of tube exposure after PMS surgery in a 59-year-old woman (9). Fahy et al. reported a case of tube exposure 8 weeks after needling following PMS surgery (10). The current case differs from these previous reports of distal tube exposure in that the conjunctiva overlying the microshunt was linearly perforated. Regarding PMS tube perforation, only one case has been reported, in which the tube was transected during bleb revision 5 months postoperatively (11).

Although the exact mechanisms that led to the shunt exposure and perforation have not been clarified, we hypothesize the following mechanisms. First, the conjunctiva overlying the microshunt underwent linear perforation. The mechanism that led to the conjunctival perforation may be the thinning and the mechanical failure of the conjunctiva and Tenon’s capsule due to the combined effects of multiple surgeries and the toxicities associated with long-term exposure to topical medications and preservatives. Bunod et al. reported that intraoperative defects in the Tenon’s capsule are a risk factor for conjunctival exposure of the tube (2). The patient had used glaucoma eye drops long-term, containing benzalkonium chloride, which is known to cause conjunctival inflammation (12). Prostaglandin-associated periorbitopathy (PAP) may have further predisposed the conjunctiva to perforation in this patient. PAP is caused by prostaglandin analogs and includes skin hyperpigmentation, eyelash elongation, dermatochalasis involution, and DUES, along with loss of lower lid steatoblepharon, upper lid ptosis, lower lid retraction, and enophthalmos (13–15). The eyelids of patients with PAP are known for their increased rigidity (16). Repeated mechanical friction from these hardened lids likely compromised the conjunctiva, as it was compressed between the eyelid and the underlying microshunt.

We suspect that the conjunctival break initiated a sequence in which the exposed shunt surface suffered structural compromise due to direct mechanical friction from the eyelid and interaction with the tear film.

There have been no reports of perforation of the PMS placed within the subconjunctival space. However, when the protective conjunctival barrier is lost, the microshunt is subjected to repeated direct friction from the hardened eyelid, which may compromise its structural integrity. In addition, exposure to tears and topical medications may further compromise the structural integrity of the PMS. For example, exposure to lipids has been shown to decrease the material’s physical strength (17). Additionally, according to a study reported by Tomita et al., direct contact between a petrolatum-based ophthalmic ointment and an SIBS-based MicroShunt induced swelling, compromising its structural integrity (18). In this case, a petrolatum-based ophthalmic ointment was added after conjunctival perforation, which may have compromised the structural integrity of the PMS. This case report suggests that mechanical or chemical failure of the PMS may occur when the natural protection provided by a healthy conjunctiva is lost.

In summary, tissue thinning and PAP may create an unhealthy peri-implant environment, leading to device exposure. Furthermore, the use of petrolatum-based ophthalmic ointment after tube exposure may compromise the tube’s integrity, ultimately resulting in perforation. This case underscores the importance of carefully evaluating the conjunctival health and eyelid anatomy in patients before PMS implantation. In addition, if postoperative tube exposure occurs, the use of petrolatum-based ophthalmic ointment should be avoided.

## 4 Conclusion

We reported a case of PMS exposure and tube perforation. PMS implantation is associated with few postoperative complications or interventions, but in cases in which the conjunctival condition is poor preoperatively, tube exposure and perforation may result. This case highlights the need for careful preoperative assessment of the ocular surface and vigilant postoperative monitoring for possible conjunctival breakdown and tube exposure, especially in patients with unhealthy conjunctivae. In addition, if tube exposure occurs postoperatively, the use of petrolatum-based ophthalmic ointment should be avoided because it may compromise the integrity of the tube.

## Data Availability

The original contributions presented in the study are included in the article/supplementary material. Further inquiries can be directed to the corresponding author.
